# Momentary versus Retrospective Sexual Consent Perceptions

**DOI:** 10.1007/s10508-021-02182-7

**Published:** 2021-12-01

**Authors:** Malachi Willis, Kristen N. Jozkowski

**Affiliations:** 1 Institute of Health and Wellbeing, University of Glasgow, Glasgow, G12 8QQ UK; 2 Department of Applied Health Science, School of Public Health, and the Kinsey Institute for Research in Sex, Gender, and Reproduction, Indiana University, Bloomington, IN USA

**Keywords:** Sexual consent, Hindsight bias, Affirmative consent

## Abstract

Perceiving potential indicators of a person’s willingness is an integral component of sexual consent. Preliminary qualitative evidence using vignettes suggested that consent perceptions can change over the course of a sexual scenario. In the present study, we extended previous research by directly comparing momentary and retrospective sexual consent perceptions using a quantitative study design. Employing a staggered vignette protocol, we examined participants’ (*n* = 962; 72.0% female) momentary perceptions of fictional characters’ sexual consent and compared them with participants’ retrospective perceptions of the characters’ consent. We hypothesized that participants would demonstrate a hindsight bias in that they would retrospectively indicate they thought the fictional characters were first willing to engage in sexual behavior earlier than when they did momentarily. We found that differences in participants’ momentary versus retrospective perceptions of characters’ sexual consent varied by the type of behavior. As we expected, participants demonstrated a hindsight bias for making out. Contrary to our hypothesis, participants were hesitant to retrospectively report that the characters were willing to engage in the other sexual behaviors (e.g., oral, vaginal, anal sex) at a point earlier than their momentary perceptions. That momentary and retrospective sexual consent perceptions significantly differ corroborates previous recommendations that sexual consent be conceptualized as an ongoing process.

## Introduction

Based on previous research, we defined sexual consent as one’s “willingness to engage in a particular sexual behavior with a particular person within a particular context” (Willis & Jozkowski, 2019, p. 1723). This internal state of willingness can be communicated to others in an act of agreement (Jozkowski et al., 2014). These two aspects of sexual consent—internal and external—are complemented by a third important component: sexual consent perceptions (Muehlenhard et al., 2016). In other words, sexual consent can also be conceptualized as behaviors or contexts that a person uses to infer somebody else’s willingness to engage in sexual activity.

Sexual consent perceptions have received less empirical attention than internal consent feelings or external consent communication; however, academic interest in this conceptualization of sexual consent is growing. In one study, women reported the types of cues they perceived their partner had used to communicate being willing to engage in their most recent sexual encounter (Willis, Hunt, et al., 2019; Willis et al., 2021). If they perceived their partner’s consent communication to be passive, women in that study reported elevated levels of their internal consent. In a qualitative study, Beres (2010) asked participants how they infer their partner’s willingness to engage in casual sex. Participants in that study identified specific and subtle actions (e.g., pushing into a partner, sighing, or moaning), as well as certain contexts (e.g., being alone in a private setting), that they use to perceive another person’s sexual consent. Using daily diaries, Willis and Jozkowski (2019) asked participants how they knew their partnered sexual behavior on a given day was consensual. Similar to Beres (2010), these researchers found that participants relied on both communication and context cues to perceive that a sexual encounter had been consensual.

In addition to self-reported perceptions of people’s own sexual encounters, researchers have studied sexual consent perceptions using fictional vignettes. This type of methodology allows researchers to reduce potential recall biases by capturing consent perceptions immediately after the fictional sexual encounter happens and standardize, or even manipulate, cues that participants might use to inform their sexual consent perceptions. In one study, Humphreys (2007) manipulated the relationship context between the characters (i.e., first date and never having been sexual vs. dating three months and being sexual occasionally vs. married two years and being sexual regularly) and found that it influenced perceptions of sexual consent in scenarios where consent was purposely ambiguous. Even though the potential consent communication cues—all of which were nonverbal—presented in each condition were exactly the same, scenarios that indicated a more intimate relationship with a partner were perceived as clearer in sexual intent, more acceptable, less in need of additional precautions, and overall more consensual (Humphreys, 2007).

A more recent study similarly used a vignette to depict a sexual encounter between two heterosexual characters that was purposely ambiguous regarding sexual consent (i.e., no explicit expressions of willingness were included in the vignette; Groggel et al., 2021). Participants in that study were more likely to perceive that the male character had give sexual consent compared with the female character. When asked to provide evidence supporting their sexual consent perceptions, participants identified implicit cues, such as the characters transitioning together from a public to a private setting (Groggel et al., 2021). These vignette studies presented the entire vignette before participants assessed whether the characters were willing to engage in sexual activity. As such, previous research on fictional sexual encounters has typically relied on retrospective perceptions of characters’ sexual consent.

However, there is initial evidence that people may perceive sexual consent differently in the moments that a sexual encounter unfolds than they do after knowing the outcome. Specifically, Jozkowski (2015) conducted a qualitative study that separated a vignette into five segments, which were “specifically written to include examples of determinants of sexual assault” (p. 7). After each segment was read to participants, Jozkowski (2015) asked participants (*n* = 20) to describe what was happening in the scene and what they perceived each character had been thinking during a particular vignette segment (i.e., momentary perceptions). Then after all five vignettes had been presented, participants were asked to provide an overall reflection (i.e., retrospective perceptions). Emerging themes for momentary perceptions in the earlier vignette segments included gender stereotypes and misinterpretation of nonverbal cues. However, once participants knew the outcome of the vignette and acknowledged nonconsensual sexual activity had occurred, participants retrospectively endorsed victim-blaming after all scenes had been heard. Participants even used information from the earlier vignette segments to retroactively support their perceptions of the situation after they had identified it as nonconsensual. Therefore, even though Jozkowski (2015) intended to evaluate two different approaches to sexual assault prevention rather than explicitly examine sexual consent perceptions, that study provided preliminary evidence that sexual consent perceptions might be influenced by hindsight bias.

Hindsight bias is the tendency for people with knowledge about an outcome to falsely believe that they would have predicted the outcome of an event (Hawkins & Hastie, 1990). This bias is a robust effect across studies, consistently distorting people’s memory, their belief about the likelihood of an event, and their confidence in their predictive ability (Roese & Vohs, 2012). In the case of sexual consent perceptions, Jozkowski (2015) found that cues participants had initially interpreted as potential indicators of sexual consent were later used to support perceptions that the fictional sexual encounter had been nonconsensual (i.e., after evidence had been provided that one of the characters refused sexual activity).

### Present Study

In the present study, we aimed to extend Jozkowski’s (2015) work on momentary perceptions of a sexual encounter in several ways. First, Jozkowski’s (2015) study was a qualitative assessment of 20 participants’ perceptions. We sought to complement their findings with a quantitative investigation of perceptions in a much larger sample. Second, Jozkowski’s (2015) vignette focused on a sexual encounter that was designed to be perceived as nonconsensual. Adopting Jozkowski’s (2015) staggered vignette protocol, we developed a scenario that depicted a consensual sexual encounter. Understanding perceptions of consensual sexual activity is important because previous research has demonstrated internal consent feelings and external consent communication vary even across sexual encounters identified as consensual (Willis et al., 2021). Finally, Jozkowski (2015) did not directly measure sexual consent perceptions; rather, generic prompts guided their one-on-one interviews. We planned to ask participants whether they perceived—both momentarily and retrospectively—the characters in the vignette to be willing to engage in various sexual behaviors.

Investigating people’s momentary consent perceptions may have important implications for the validity of retrospective research on sexual consent. For example, if people are relatively more apt—or more hesitant—to perceive another’s willingness to engage in sexual activity when the sexual encounter is ongoing versus having already happened, then retrospective reports may not be accurate representations of consent as a process and may instead encourage people to rely on a more discrete conceptualization of consent when responding. Because extant studies on sexual consent perceptions have relied almost exclusively on retrospective reports in which the outcome of the sexual encounter in question—fictional or otherwise—was known to the participant when they participated in the study, the need to research how aligned such retrospective reports are with momentary ones remains.

Thus, the purpose of the present study was to examine whether participants’ momentary consent perceptions differed from their retrospective reports for the same fictional sexual encounter. We predicted that participants would demonstrate a hindsight bias. Specifically, we hypothesized that participants—after learning that the fictional sexual encounter was seemingly consensual throughout the entire vignette—would retrospectively indicate they thought the fictional characters were first willing to engage in a sexual behavior earlier than when they first indicated this momentarily. In other words, we expected participants to think they knew all along that the fictional characters were willing to engage in sexual behavior.

## Method

### Participants

In sum, 1464 people started this study; 501 participants were removed for missing any data and one for being 17 years old. Thus, our analytic sample comprised 962 people. Participants were 24.4 years old on average (SD = 9.9; range = 18–79), and 80.7% (*n* = 777) were university students. Most participants identified as female (*n* = 693; 72.0%). Females tended to be exclusively or predominantly sexually attracted to males (*n* = 646; 93.4%); males tended to be exclusively or predominantly sexually attracted to females (*n* = 227; 84.7%). About half of the participants were in a committed relationship at the time of the study (*n* = 460; 48.0%). Regarding racial/ethnic identity, 83.1% identified as White (*n* = 799), 8.1% as Black (*n* = 78), 5.4% as Hispanic (*n* = 52), and 4.3% as Asian (*n* = 41).

### Material

To examine participants’ momentary perceptions of characters’ sexual consent, we developed a staggered vignette protocol. In other words, we presented a limited amount of information to participants regarding a fictional sexual encounter between two characters. We then asked participants questions regarding the characters’ sexual consent before presenting them with further information. In this way, we were able to capture participants’ perceptions of the characters’ consent as they were reading about the development of the sexual encounter, which ultimately turned out to be consensual. Finally, we examined consent perceptions as a function of two constructs that are related to sexual consent communication: gender of the character and type of sexual behavior (Humphreys, 2007; Willis, Hunt, et al., 2019).

To examine people’s momentary consent perceptions, we developed a vignette of a fictional consensual sexual encounter between a woman (Kim) and a man (Mike) and presented information in a staggered manner (see Appendix). We included three manipulations with two levels each, resulting in eight experimental conditions. Specifically, we counterbalanced the gender of the characters (i.e., alternated the names across conditions so no consent cue was systematically associated with one gender), manipulated whether one of the characters accepted a drink from the other, and manipulated whether the characters had just met or had been friends for a few months. Participants were randomly assigned to one of these conditions.

In 11 segments, our vignette depicted these two fictional characters progressing from flirting in a public setting at the start of the vignette to engaging in sexual behavior in a private setting at the end of the vignette. Informed by previous research, we included a variety of nonverbal sexual consent cues across all segments of the vignette (Beres, 2010; Hickman & Muehlenhard, 1999; Jozkowski et al., 2018; Marcantonio et al., 2018). Implicit nonverbal consent cues in the vignette included touching somebody’s hand and arm and smiling; explicit nonverbal consent cues included lifting hips for somebody to take off underwear and presenting a condom.

We piloted this vignette with graduate and undergraduate research assistants to improve its believability. In the present study, 83.7% of participants indicated that they thought the vignette was *Extremely believable* or *Moderately believable*; only 2.9% indicated that they thought the vignette was *Extremely unbelievable* or *Moderately unbelievable*.

### Procedure

These data are part of a larger study that developed a staggered vignette protocol (Willis & Jozkowski, 2021). Vignettes have been used in behavioral, social, and psychological research to examine various phenomena (e.g., Aguinis & Bradley, 2014; Steiner et al., 2016); Jozkowski (2015) also used a staggered vignette to examine sexual consent specifically.

Anybody over the age of 18 was eligible to participate. Participants were recruited via social media, word-of-mouth, or instructors at a large public university in the southern United States; those in university courses were offered course credit for their participation. Interested people accessed the study online via Qualtrics Survey Software. All study procedures were approved by the university’s institutional review board.

After first filling out sociodemographic items, participants were presented a randomly assigned vignette that described a sexual encounter. The vignette was presented in 11 discrete segments. After each segment, participants indicated how willing they thought each character was to engage in sexual behaviors (i.e., making out, genital touching, oral sex, vaginal sex, and anal sex). Once participants completed all 11 segments, they were asked to retrospectively indicate the point at which they perceived each character to first be willing to engage in each of the sexual behaviors.

### Measures

#### Sociodemographic Variables

Participants reported their identity regarding a variety of sociodemographic variables: age, race/ethnicity, sex, sexual attraction, relationship status, and university status.

#### Momentary Consent Perceptions

To measure participants’ momentary consent perceptions, we asked them after each of the 11 vignette segments, “From the information provided, do you think [Kim/Mike] would be willing to engage in any of the following behaviors with [Mike/Kim]?” Participants were randomly assigned to report their consent perceptions for Kim first or Mike first. They responded on a seven-point Likert-type scale: *Definitely not*, *No*, *Probably not*, *Not sure*, *Probably*, *Yes*, *Definitely*. Based on these ratings, we created a variable that indicated the segment of the vignette at which participants first perceived a character to be willing to engage in a sexual behavior (i.e., the first time they responded *Yes* or *Definitely*). Higher scores indicate that participants first selected *Yes* or *Definitely* at a segment that appeared later in the vignette.

Because sexual consent behaviors and perceptions can vary by the type of sexual behavior (Humphreys, 2007; Marcantonio et al., 2018; Willis, Hunt, et al., 2019), we asked participants to report their consent perceptions of the characters for five different behaviors: making out, genital touching, oral sex, vaginal sex, and anal sex.

#### Retrospective Consent Perceptions

After presenting all 11 segments of the vignette and asking participants’ momentary consent perceptions, we asked participants to indicate the point at which they thought the characters were willing to engage in each of the same five sexual behaviors with each other. The response options included each of the 11 vignette segments as well as a twelfth option: “I don’t think [Kim/Mike] was ever willing to [insert sexual behavior] with [Mike/Kim].” Higher scores indicate that participants first thought the characters were willing at a segment that appeared later in the vignette. If participants indicated retrospectively that they did not think a character was willing to engage in a sexual behavior at any point in the vignette, they were coded as missing for this variable.

### Analysis

To examine whether people’s momentary perceptions of sexual consent differed from their retrospective perceptions, we conducted a four-way ANCOVA. Sexual consent perceptions were the dependent variable. Within-person independent variables included timing of report (momentary versus retrospective), type of sexual behavior^1^ (making out, genital touching, oral sex, and vaginal sex), and gender of the character (female versus male). Gender of the participant was included as a between-person independent variable, and the three experimental conditions were included as covariates in the model because these manipulations were not relevant to the present study’s research question.

To test simple mean effects, we used Helmert contrasts, which allowed us to rank order the types of sexual behavior. Specifically, we compared effects for one level with all higher-order levels. To control for Type 1 error rates, we used a Bonferroni correction; all tests had an *α*-level of 0.0045 because the ANCOVA model included 11 independent variables. Analyses were conducted with SPSS 26.

## Results

### Descriptive Statistics

After reading only the first segment of the vignette, only 32.3% of participants reported that at least one of the characters was willing to engage in making out—18.9% in genital touching, 16.0% in oral sex, and 15.1% in vaginal sex. After reading the entire vignette, 31.5% of participants retrospectively reported that at least one of the characters was willing to engage in making out as early as the first segment of the vignette—9.7% for genital touching, 8.4% for oral sex, and 8.4% for vaginal sex. Thus, there was descriptive evidence that participants’ momentary perceptions typically differed from their retrospective perceptions from the start of the vignette.

As the vignette progressed, people were more likely to indicate that the characters would be willing to engage in sexual activity. For example, halfway through the vignette, 96.6% of participants reported that at least one of the characters was willing to engage in making out. The proportions also increased to 82.1% for genital touching, 63.7% for oral sex, and 61.5% for vaginal sex. Retrospectively, 99.4% of participants reported that at least one of the characters was willing to engage in making out at the sixth or earlier segment of the vignette—85.9% for genital touching, 50.2% for oral sex, and 42.7% for vaginal sex.

### ANCOVA Model

We tested an ANCOVA model to examine whether the differences between momentary and retrospective sexual consent perceptions were significant even after controlling for other relevant variables. See Table 1 for a comprehensive presentation of sexual consent perceptions by within-person factors.

**Table 1:** Descriptive Statistics of Sexual Consent Perceptions (N = 962)

Sexual behavior	Character gender	Momentary		Retrospective	
		*M*	SD	*M*	*SD*
Making out	Female	3.35	1.99	2.92	1.57
	Male	3.12	1.89	2.70	1.68
Genital touching	Female	4.74	2.46	5.14	2.00
	Male	4.50	2.42	4.85	2.16
Oral sex	Female	5.84	2.94	6.90	2.35
	Male	5.57	2.93	6.61	2.57
Vaginal sex	Female	6.15	3.08	7.59	2.74
	Male	5.80	2.99	7.12	2.84

Values reflect the vignette segment that people first indicated—momentarily or retrospectively—that they perceived the fictional character to be willing to engage in the sexual behavior. Higher scores indicate that participants first thought the characters were willing at a segment that appeared later in the vignette. Absolute range: 1 to 11

#### Within-Person Effects

The point at which people perceived the characters to be willing to engage in sexual behavior significantly differed based on whether they were providing momentary or retrospective reports and controlling for all other variables in the model, *F*(1, 957) = 15.38, *p* < .001, partial η^2^ = 0.02. Sexual consent perceptions varied to an even greater extent as a function of the type of sexual behavior, *F*(3, 957) = 645.14, *p* < .001, partial *η*^2^ = 0.40, and the gender of the character, *F*(1, 957) = 368.70, *p* < .001, partial *η*^2^ = 0.28 (see Table 2).

**Table 2:** ANCOVA predicting sexual consent perceptions

	MS_E_	*df*	*F*	*p*	η^2^
*Within-person*					
Timing	216.13	1	15.38*	< .001	.016
Sexual behavior	2207.76	3	645.14*	< .001	.403
Character gender	2314.43	1	368.70*	< .001	.278
Timing X sexual behavior	111.17	3	54.92*	< .001	.054
Timing X character gender	2.55	1	1.25	.264	.001
Sexual behavior X character gender	60.28	1	73.69*	< .001	.071
Timing X sexual behavior X character gender	1.80	3	2.73	.043	.003
*Between-person*					
Participant gender	220.65	1	9.20*	< .001	.010
Condition (Order)	164.64	1	6.87	.009	.007
Condition (relationship)	91.42	1	3.81	.051	.004
Condition (alcohol)	2.44	1	0.10	.750	.000
*Residual*	23.98	957			

MS_E_ = mean square error. *Statistically significant once accounting for the Bonferroni correction (i.e., *α* = .0045)

The effect of timing on sexual consent perceptions was significantly moderated by type of sexual behavior, controlling for all other variables in the model, *F*(3, 957) = 54.92, *p* < .001, partial *η*^2^ = 0.054. Compared with relatively higher-order sexual behaviors, the effect of timing was significantly different for making out, *F*(1, 957) = 81.57, *p* < .001, partial η^2^ = 0.08, and genital touching, *F*(1, 957) = 50.87, *p* < .001, partial η^2^ = 0.05. Further, the effect of timing significantly differed—but to a lesser extent—between oral sex and vaginal sex, *F*(1, 957) = 7.25, *p* = .007, partial *η*^2^ = 0.01.

**Fig. 1:**
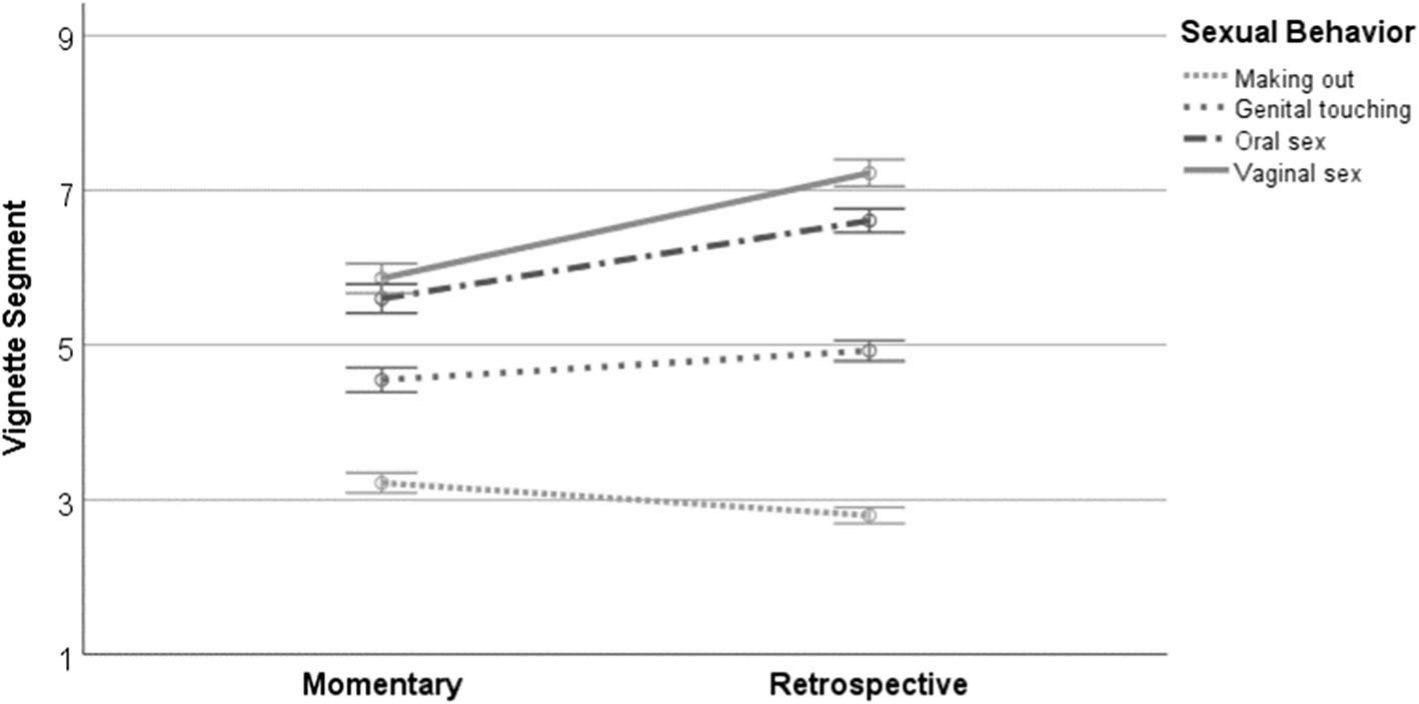
Simple mean effects depicting the interaction between timing of reports and type of sexual behavior. Plotted means were adjusted based on the covariates included in the hypothesized model. Error bars represent 95% confidence intervals

Figure 1 depicts the directions of the simple effects that composed the interaction between timing and type of sexual behavior. Participants’ momentary consent perceptions for making out (*M*_adj._ = 3.22) were greater than their retrospective consent perceptions (*M*_adj._ = 2.80), which indicated that they thought the characters were willing to engage in this behavior at a relatively earlier point in the vignette once they had read the entire vignette. However, this effect was reversed for the other sexual behaviors; momentary consent perceptions for genital touching (*M*_adj._ = 4.55), oral sex (*M*_adj._ = 5.60), and vaginal sex (*M*_adj._ = 5.86) were lower than their retrospective consent perceptions (*M*_adj._ = 4.92, 6.61, 7.22, respectively).

There was not a significant interaction between timing and character’s gender in their effects of sexual consent perceptions, controlling for all other variables in the model, *F*(1, 957) = 1.25, *p* = .264, partial η^2^ < 0.01. In other words, participants’ retrospective consent perceptions consistently differed from their momentary consent perceptions disregarding the character’s gender. Additionally, the significant interaction between timing and type of sexual behavior was not significantly moderated by character’s gender once accounting for the Bonferroni correction, *F*(3, 957) = 2.73, *p* = .043, partial η^2^ < 0.01.

#### Between-Person Effects

Gender of the participant was associated with sexual consent perceptions, *F*(1, 957) = 9.20, *p* < .001, partial η^2^ = 0.010 but did not moderate the effect of timing, *F*(1, 957) = 0.29, *p* = .590, partial η^2^ < 0.01. As covariates, we also included the three experimental manipulations in the model; however, sexual consent perceptions did not significantly differ across any of these conditions once accounting for the Bonferroni correction, *F*s(1, 957) ≤ 6.87, *p*s ≥ 0.009, partial η^2^s ≤ 0.01.

## Discussion

Alongside feelings and communication, perceptions constitute one of the three core conceptualizations of sexual consent (Muehlenhard et al., 2016). Previous research has used vignettes to assess people’s consent perceptions; such studies typically presented a fictional sexual encounter to participants, who were then asked to retrospectively report their perceptions (Groggell et al., 2021; Humphreys, 2007). To our knowledge, only one study has used a staggered vignette protocol to assess sexual consent perceptions (Jozkowski, 2015). Using a qualitative study design, Jozkowski (2015) provided preliminary evidence that sexual consent perceptions change as more information is provided to participants. We sought to complement Jozkowski’s (2015) findings by conducting a quantitative study to directly examine sexual consent perceptions that were measured momentarily versus retrospectively with a much larger convenience sample.

Controlling for key constructs related to sexual consent (i.e., gender and type of sexual behavior), we found that the point at which participants perceived the fictional sexual encounter to be consensual when they were reading the vignette one segment at a time (i.e., momentary perceptions) varied from the point they identified after having read the entire vignette (i.e., retrospective perceptions). We expected that people—once knowing that the sexual encounter was seemingly consensual—would retrospectively indicate consent earlier than their momentary responses, providing evidence for a hindsight bias. However, the direction of this difference significantly varied by the type of sexual behavior in question.

Participants only demonstrated a hindsight bias for one behavior; specifically, they retrospectively perceived characters were willing to engage in making out at an earlier point in the vignette than they had indicated at the moment. Having witnessed the characters eventually engage in seemingly consensual higher-order behaviors, participants may have believed the characters were initially willing to make out even though they had not thought so before knowing the outcome of the scenario. In this way, participants may have reevaluated the sexual encounter once they were able to reflect on all the information.

Contrasting our hypothesis that participants’ perceptions would align with literature on hindsight bias, this pattern was reversed for other sexual behaviors we assessed (i.e., genital touching, oral sex, vaginal sex). Rather than reporting they knew all along that the characters were willing to engage in these sexual behaviors, participants’ retrospective perceptions demonstrated a hesitancy effect. In other words, participants did not perceive sexual consent as early as they had at the time they were witnessing the encounter unfold. This effect did not vary by the character’s gender, but it increased with the intimacy of the sexual behavior. People were retrospectively more hesitant to perceive a character’s sexual consent for vaginal sex than oral sex—and were more hesitant for oral sex than genital touching.

That participants were more hesitant to indicate after the fact that the characters were willing to engage in these sexual behaviors for this particular vignette was curious because we exclusively provided cues most people interpret as indicating consent—several of which were explicit. We expected people to retrospectively perceive the characters’ consent earlier than they had at the moment for all behaviors. One potential explanation for this unexpected finding is that people may rely on more explicit cues or cues that are more proximal to the sexual encounter to indicate sexual consent when they are available. Jozkowski and colleagues (2018) argued that certain cues—perhaps those that did not happen immediately before the sexual behavior—may seem less important to people hearing about a sexual event after the fact. If this is the case, our findings would be important for research on the process of consent in sexual relationships. By asking people to report the consent cues that they remember, research that uses retrospective data may not capture cues that participants either forget or disregard once something more convincing happens. Therefore, retrospective reports regarding sexual consent may be subject to memory biases.

Another possible explanation for why participants did not demonstrate a hindsight bias is that they succumbed to a social desirability bias, which refers to instances wherein participants attempt to endorse the most socially acceptable position instead of the position that most accurately reflects their beliefs (DeMaio, 1984). Researchers have previously proposed that momentary reports are less vulnerable to social desirability bias and more likely to capture “undesirable” thoughts and feelings compared with retrospective reports (Blome & Augustin, 2015; Schwarz, 2007). Specifically, it may be more salient to participants that they are providing an evaluation when asked to reflect and provide their retrospective perceptions of an entire scenario—rather than when they are asked to provide seemingly quick momentary perceptions of limited information. Regarding the instance of hindsight bias that we found for making out, it might be that people think it is more socially acceptable to assume a person’s sexual consent to this sexual behavior; as such, they would not demonstrate a potential social desirability bias for this behavior. However, for more intimate behaviors (e.g., vaginal sex), people may have identified the more explicit cues that occurred later in the vignette as indicating sexual consent as a function of social desirability. Social desirability might have been particularly relevant in our sample of mostly university students, who may have received on-campus affirmative consent education.

### Implications

In the moment, people seem to rely on relatively less explicit cues to assume somebody’s willingness to engage in sexual activity than they use to retroactively justify consent. Thus, the cues people use to perceive sexual consent may vary over the course of a sexual encounter and even afterward—corroborating previous suggestions that sexual consent is theoretically fluid and fluctuates with the ever-changing context of a particular sexual encounter (Willis & Jozkowski, 2019). Our findings are also in line with research demonstrating that people use subtle cues to infer another person’s willingness to engage in sexual activity and that they might overreport their reliance on explicit cues (Beres, 2010). Taken together, the importance of multiple cues—potentially implicit ones—in the developmental process of a consensual sexual encounter should be acknowledged.

Sexual consent does not transition from being absent to present with a single action (e.g., asking somebody to have sex). As our findings show, people may indeed look for a discrete indicator of willingness when they retrospectively evaluate a sexual encounter. However, momentary perceptions seem less distinct and instead change as new information is provided. Therefore, the present study corroborates previous conceptualizations that sexual consent is an ongoing and iterative process that builds toward and continues throughout a consensual sexual encounter (Beres, 2010; Humphreys, 2004; Muehlenhard et al., 2016). No single cue guarantees a person’s sustained willingness to engage in a particular sexual behavior with a particular person. As a situation changes, so might sexual consent. As such, people should actively be aware of their partner’s cues in the moment and not slide into assuming sexual consent based on previous cues—no matter how recently they occurred.

We encourage those implementing sexual consent education programs, especially affirmative consent or “yes means yes” initiatives, to emphasize in their curricula that sexual consent does not stop once another person has agreed to engage in a particular sexual activity within a particular context. In line with recommendations provided in previous research, such programs should seek to describe sexual consent as a process whereby perceiving another person’s agreement—no matter how explicit or verbal their cues are—at one point in time does not imply agreement nor obligation to engage in sexual activity at a later point in time (Muehlenhard et al., 2016; Willis & Jozkowski, 2019).

While the present study’s implications regarding nonconsensual sexual activity are limited due to the fact that our vignette was purposely designed to be unambiguously consensual, preliminary insight and speculations can be gleaned from our findings. If future researchers investigated people’s consent perceptions using a staggered vignette of a sexual encounter that eventually becomes nonconsensual, they might find that people would perceive the beginning interactions leading to a potential sexual encounter to be okay at the moment; however, if it became clear that one of the characters was not willing to engage in sexual activity, then people’s retrospective perceptions of the situation would likely contradict the momentary perceptions (Jozkowski, 2015). This speculated finding could be useful in allaying some of the questions people pose regarding why victims stayed in situations in the moment but only retrospectively identified the encounter as not okay or unsafe. Further examination is needed to substantiate this possibility.

### Limitations

Our findings should be carefully considered in light of several limitations to the present study. These limitations provide avenues for future research.

First, consistent with most vignette studies, our scenario was limited in how applicable it might have been for individual participants. While using a vignette that depicted a single set of consent cues allowed us to systematically control for the evidence participants could use to inform their consent perceptions, human sexual behavior is diverse and cannot be adequately represented by a monolith—despite our efforts to develop a fictional sexual scenario that could be generalizable to many people. Future research using vignettes to investigate sexual consent should consider manipulating various aspects of the story to better capture people’s diverse sexual experiences (e.g., types of consent or refusal communications, the contexts or circumstances underlying the sexual encounter, the individual differences of the characters).

Second, our primarily White and female sample was limited in its generalizability to more diverse populations. While the average age of our participants remained younger, a strength of our study was that the data did not only reflect the sexual consent perceptions of students; 85% of previous empirical work on sexual consent has used samples that exclusively comprised students (Willis, Blunt-Vinti, & Jozkowski, 2019).^2^ Future work on sexual consent should rely less on convenience sampling to collect data from samples that are more diverse regarding characteristics such as age, race/ethnicity, gender identity, sexual orientation, ability, and so on.

Third, the measures used in this study were not subjected to rigorous validation processes. While our measurement decisions were informed by previous research, we did not ground the development of our items measuring sexual consent in psychometric assessment. Future research should consider critically assessing the face validity, content validity, and construct validity of these measures before administering them. Further, we did not include attention checks in our survey; doing so would help identify low-quality responses.

### Conclusion

In the present study, we used a staggered vignette protocol to measure momentary sexual consent perceptions and compare them with retrospective perceptions. We provided evidence that people’s sexual consent perceptions changed once they were able to reflect on an entire sexual encounter; the direction of this difference varied by the type of the behavior. That momentary and retrospective consent perceptions vary indicates the importance of conceptualizing sexual consent as an ongoing process. Sexual partners should remain attentive to each other’s consent communication in the moment.

## Appendix

Example vignette script

1. Mike and Kim have been friends for a few weeks. Tonight, they are hanging out at a bar and are enjoying each other's company. Mike offers Kim a drink. Kim says she’s not drinking tonight.
2. Both are flirting with each other. Kim touches Mike’s arm while she laughs at something he says.
3. Mike invites Kim to get a ride home with him. Kim accepts.
4. Once at Mike’s place, they decide to watch a movie together. Mike starts the movie and then sits down next to Kim so that their legs are touching.
5. A few minutes into the movie, Kim reaches for Mike’s hand. Mike smiles and places his hand in hers.
6. A while later, Mike leans in to kiss Kim. They start making out. Mike reaches his hand under Kim’s shirt. Kim pulls him closer.
7. They take off their own shirts, and Mike undoes Kim’s bra. Mike then leads Kim to his bedroom. Kim follows.
8. They both take off their own pants before getting into Mike’s bed. Mike and Kim start making out again, Mike on top.
9. Mike kisses Kim’s neck and continues to do so from her breasts to her stomach. Kim lifts her butt for Mike to take off her underwear.
10. Mike takes off Kim’s underwear and starts to go down on her. After a while, Mike crawls up to his nightstand and grabs a condom.
11. Kim takes the condom, opens it, and puts it on Mike. Mike and Kim begin to have sex.

## Footnotes

1 We did not include sexual consent perceptions for anal sex in the model because only 13.1% (*n* = 126) of participants perceived the characters as willing to engage in this sexual behavior at any point of the vignette.

2 See Willis and Jozkowski (2021) for a comparison of sexual consent perceptions between students versus non-students.
